# Optimization of Mix Design for Lightweight Boards Based on GGBFS–Waste Rock Wool Using Response Surface Methodology

**DOI:** 10.3390/ma18235376

**Published:** 2025-11-28

**Authors:** Jun-Cheol Lee

**Affiliations:** Department of Architecture, Seowon University, Cheongju 28674, Republic of Korea; leejc@seowon.ac.kr; Tel.: +82-43-299-8788

**Keywords:** response surface methodology, optimal mix design, lightweight board, ground granulated blast-furnace slag, waste rock wool

## Abstract

This study aimed to establish the optimal mix proportions for eco-friendly lightweight boards based on Ground Granulated Blast-furnace Slag (GGBFS) and waste rock wool using Response Surface Methodology (RSM). The investigation focused on optimizing three key properties: flexural failure load (*Y*_1_), moisture content (*Y*_2_), and specific gravity (*Y*_3_). ANOVA results identified Binder and Perlite as the most dominant and statistically significant factors, exhibiting critical conflicting effects necessary for balancing strength and lightweight goals. Wollastonite showed a non-linear effect on flexural strength, peaking at an intermediate level. A Response Optimization simulation, targeting a minimum flexural load of 400 N, moisture content of 2.0%, and specific gravity of 0.80, yielded an optimal mix proportion: Binder 52.12%, Perlite 48.45%, and Wollastonite 7.37%. This blend achieved a high Composite Desirability (D) of 0.8725. Experimental verification confirmed the model’s reliability. The measured flexural load (408.54 N) successfully exceeded the 400 N target, and all measured values exhibited a low error margin (under 7%) compared to the predicted values. This optimized mix proportion provides a reliable foundation for developing high-performance, sustainable lightweight construction materials.

## 1. Introduction

The modern construction industry, driven by contemporary demands for climate change mitigation and resource circulation, prioritizes enhancing energy efficiency and reducing environmental burdens [1,2,3]. Consequently, there is a strong need for the development of eco-friendly building materials. In particular, the demand for lightweight boards, which offer superior thermal insulation, fire resistance, and construction convenience, is consistently rising, aligning with the trend towards structural weight reduction in buildings [4,5,6]. However, conventional lightweight board products often involve high-temperature firing processes or rely on expensive natural raw materials during manufacturing, resulting in high production costs and significant environmental loads [7,8]. To overcome these limitations and develop sustainable construction materials, the establishment of an economic and environmentally friendly alternative material system utilizing industrial waste is urgently required [9,10,11].

This study focuses on establishing a binder system primarily composed of Ground Granulated Blast-furnace Slag (GGBFS), a major byproduct of the steelmaking process, and waste rock wool, which poses environmental disposal challenges. When activated by alkali stimulants, GGBFS and waste rock wool can be used as eco-friendly alkali-activated binders to substitute conventional cement [12,13]. Alkali-activated materials are typically classified as either two-part (requiring separate liquid activators) or one-part (where the solid alkali activator is incorporated into the dry binder powder) mixtures. The distinction is critical because the one-part, ‘just-add-water’ approach generally offers a lower carbon footprint and reduced handling risks compared to the two-part method, primarily by eliminating the energy-intensive production and transport of highly concentrated liquid alkali solutions [14,15]. These newer formulations integrate the solid activator and precursor, simplifying on-site use to only require water addition. The GGBFS-waste rock wool binder system in this study pursues this single-componentization.

To produce a high-performance, pressed lightweight board, we incorporated perlite [16] as a main filler to ensure lightness, wollastonite [17], which possesses an acicular structure for strength reinforcement, and cellulose reinforcement [18] to enhance board toughness.

The performance of such multi-component composite material systems—including key properties like flexural strength, moisture content, and apparent specific gravity—is highly complex and interdependent on the content of each constituent [19]. Therefore, traditional experimental methods that vary only a single factor make it difficult to identify the interactions between factors and are limited in efficiently finding the optimal mix proportion that yields the best performance [20,21,22].

Thus, the core objective of this study is to establish the optimal fundamental mix design for lightweight board manufacturing by adopting Response Surface Methodology (RSM) [23,24]. RSM is a statistical technique capable of quantitatively analyzing the complex effects of multiple independent variables on multiple response values with a minimal number of experiments. Specifically, binder content, perlite mixing ratio, and wollastonite mixing ratio were set as independent variables. Based on predefined target performance criteria, RSM was used to analyze the individual and interactive effects of these factors. Ultimately, the study aims to manufacture actual lightweight boards using the derived optimal mix proportion, and compare and verify the measured performance against the RSM predictions, thereby providing reliable foundational design data for the development of GGBFS-based lightweight boards and laying the groundwork for future commercialization research.

## 2. Experimental Design and Methods

In this experiment, the mixing ratios of the main raw materials—binder, perlite, and wollastonite—which significantly influence the key properties of the lightweight board, specifically flexural failure load, moisture content, and specific gravity, were set as the independent variables. To statistically analyze the complex combined effects of these factors and efficiently locate the optimum mix, the Box–Behnken Design (BBD), a subset of the Response Surface Methodology (RSM), was applied. The analysis results were utilized to derive and verify the optimal mix proportion based on target performance criteria: a flexural failure load of 400 N, a moisture content of 2.0%, and a specific gravity of 0.9.

### 2.1. Materials

The manufacturing of the lightweight board in this study utilized an eco-friendly alkali-activated binder system, with the specific mix proportions detailed in Table 1. The binder system was primarily composed of Ground Granulated Blast-furnace Slag (GGBFS) and Waste Rock Wool. For the GGBFS, Type 2, which satisfies the Korean standard KS F 2563, was used [25]. The waste rock wool, sourced from hydroponic media, was collected, washed, subjected to a calcination process at 200 °C for two hours, and then pulverized using a ball mill to achieve a fine powder with an average particle size of 7 µm. The chemical composition of the waste rock wool is presented in Table 2, with its major components including SiO_2_ (33.32%), CaO (21.24%), and Al_2_O_3_ (15.77%).

Anhydrous Gypsum was incorporated to ensure the hydration stability of the binder and promote early strength development. To induce the potential hydraulic and pozzolanic activity of the GGBFS and waste rock wool, Sodium Hydroxide (NaOH) and Sodium Carbonate (Na_2_CO_3_) were used in combination as alkali activators [12,13].

In addition to the binder materials, Perlite was used as the primary filler to secure the lightweight property of the final board. Wollastonite, characterized by an acicular structure, was utilized as a reinforcing agent to enhance flexural strength and increase the internal cohesion between materials, thereby suppressing cracking [16,17]. Furthermore, Cellulose Reinforcement was employed to boost the board’s toughness and internal binding strength, aiming to suppress brittle failure and mitigate crack generation due to shrinkage [18]. The dosage of the cellulose reinforcement was fixed at 3% of the total combined weight of the binder, perlite, and wollastonite used in the lightweight board manufacturing process.

### 2.2. Experimental Design and Statistical Analysis

The mixing ratios of binder (*X*_1_), perlite (*X*_2_), and wollastonite (*X*_3_), which were deemed to exert the most significant influence on the properties of the lightweight board, were set as the independent variables (experimental factors). For each factor, a three-level range was planned, as detailed in Table 3. The dependent variables (responses) selected were the core performance indicators of the lightweight board: Flexural Failure Load (*Y*_1_), Moisture Content (*Y*_2_), and Specific Gravity (*Y*_3_).

The statistical significance of the derived models was evaluated using a predefined significance value (α) of 0.05 (*p* < 0.05). This standard threshold is used in materials engineering to maintain an acceptable 5% risk (Type I Error) of concluding that a non-significant factor influences the product’s performance, thereby ensuring the reliability and practicality of the final model.

To efficiently predict the interactions between factors and the optimal conditions with a minimum number of experiments, the Box–Behnken Design (BBD), a type of Response Surface Methodology (RSM), was adopted. The BBD, combining three factors at three levels, resulted in a total of 15 experimental runs (12 factor points and 3 replicated center points), which were conducted in a randomized order as shown in Table 4.

Samples were prepared according to the mix proportions of the 15 experimental runs, and the flexural failure load, moisture content, and specific gravity were tested. The relationship between each dependent variable (*Y*) and the independent variables (*X_i_*) was analyzed by assuming a Second-order Polynomial Model, as represented by Equation (1):

(1) $$Y=b_{0}+\sum_{i=1}^{3}b_{i}X_{i}+\sum_{i=1}^{3}b_{ii}X_{i}^{2}+\sum_{i<j}^{3}b_{ij}X_{i}X_{j}+e\;$$

where *b*_0_ is the constant term, *b_i_* are the linear coefficients, *b_ii_* are the quadratic coefficients, *b_ij_* are the interaction coefficients, and *e* represents the error. The statistical significance (*p*-value) and coefficient of determination (R^2^) of the derived regression models were verified through Analysis of Variance (ANOVA). These models were subsequently used to visualize the response surface and perform optimization.

The verified, statistically significant second-order polynomial regression models were utilized with the Minitab 18 software to perform Response Optimizer analysis, aiming to derive the optimal mix proportion that satisfies the target performance of the lightweight board. Optimization was conducted for the three dependent variables—Flexural Failure Load (*Y*_1_), Moisture Content (*Y*_2_), and Specific Gravity (*Y*_3_)—with target values set at 400 N, 2.0%, and 0.9, respectively. During the optimization process, the incorporation level of each factor (*X*_1_, *X*_2_, *X*_3_) was explored within the response surface to determine the mix point (optimal mix proportion) that maximizes the Composite Desirability for the established target values.

The resulting optimal mix proportion was subjected to a verification experiment to confirm its practical applicability and reliability. Samples were prepared using the derived optimal mix proportion, and material tests for flexural failure load, moisture content, and specific gravity were conducted and compared with the RSM model’s Predicted Value. The predictive accuracy of the model was evaluated by calculating the percentage error between the experimental and predicted values. The optimal mix proportion was considered validated if the percentage error was within the acceptable range (±5%).

### 2.3. Manufacturing and Testing Methods for Lightweight Boards

Binder, perlite, and wollastonite were weighed according to the mix proportions for each experimental run detailed in Table 4. To prevent the fracture of the perlite particles during mixing, a two-stage low-speed mixing method was employed.

First, the binder components—GGBFS, waste rock wool, anhydrous gypsum, alkali activators, and mixing water—were introduced into a high-speed mixer and blended for approximately three minutes to produce a homogeneous paste. Subsequently, perlite, wollastonite, and the cellulose reinforcement were added. The mixer speed was then reduced, and mixing was performed for another three minutes, minimizing the shear force applied to the mixture.

**Figure 1:**
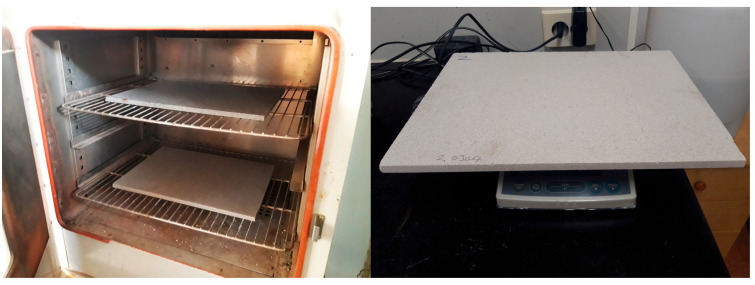
Manufactured lightweight board samples.

Two hundred grams of the finished mixture were poured into a 150 mm × 100 mm mold. The mixture was pressed under a constant pressure of 50 kg/cm^2^ to form a sample with a final thickness of 12 mm. Immediately after demolding, the molded samples underwent heat-humid curing to promote initial strength development and accelerate the alkali-activation reaction. The samples were cured for 10 h in a thermo-hygrostat chamber maintained at a temperature of 50 °C and a relative humidity of 90% or higher. Figure 1 illustrates the manufactured lightweight board samples.

The manufactured samples were subjected to flexural failure load and moisture content tests in accordance with the Korean standard KS F 3504 [26]. The specific gravity of the samples was calculated using the dry weight obtained from the moisture content test and the calculated volume of the specimens.

## 3. Experimental Results

### 3.1. Physical Property Test Results

The basic physical property test results—Flexural Failure Load (*Y*_1_), Moisture Content (*Y*_2_), and Specific Gravity (*Y*_3_)—measured for the lightweight board samples prepared according to the 15 experimental runs designed by the Box–Behnken Design (BBD) are presented in Table 5.

The flexural failure load measurements ranged widely, from a minimum of 260.0 N (Run No. 15) to a maximum of 479.7 N (Run No. 2). The highest load was recorded under conditions of maximum binder (*X*_1_ = 60%) and minimum perlite (*X*_2_ = 30%) (Run No. 2). This aligns with the general tendency for strength to increase when the proportion of the binding agent is high and the proportion of the relatively weaker lightweight filler is low. Conversely, the lowest flexural failure load (260.0 N) occurred at conditions of minimum binder (*X*_1_ = 30%) and maximum perlite (*X*_2_ = 60%) (Run No. 15).

The moisture content ranged from a minimum of 1.2% (Run No. 2) to a maximum of 4.7% (Run No. 15). The condition that exhibited the highest flexural failure load (Run No. 2) also showed the lowest moisture content, while the condition with the lowest flexural failure load (Run No. 15) resulted in the highest moisture content. This suggests a tendency for moisture content to increase as the proportion of perlite (*X*_2_) increases due to its high porosity.

The specific gravity measurements ranged from a minimum of 0.66 (Run No. 15) to a maximum of 1.21 (Run No. 2). Specific gravity was most significantly affected by the perlite (*X*_2_) content. Conditions with low perlite content (*X*_2_ = 30%) (Run Nos. 2, 6, 9) showed high specific gravity (1.08~1.21), whereas conditions with high perlite content (*X*_2_ = 60%) (Run Nos. 3, 7, 10, 14, 15) resulted in low specific gravity (0.66~0.82).

Analysis of the experimental results indicates that the change in wollastonite (*X*_3_) mixing ratio (4~10%) had a relatively marginal effect on all three physical properties compared to the drastic changes observed with the binder (*X*_1_) and perlite (*X*_2_) ratios. This suggests the necessity of verifying the statistical significance of the wollastonite factor through subsequent ANOVA analysis.

### 3.2. Response Surface Analysis Results and Statistical Significance Evaluation

#### 3.2.1. Second-Order Polynomial Regression Models

Analysis of the experimental data was performed using Minitab 18 to quantify the relationship between the three physical properties of the lightweight board (*Y*_1_, *Y*_2_, *Y*_3_) and the independent variables (*X*_1_, *X*_2_, *X*_3_). This analysis yielded specific regression models for each response based on the coded values of *X*_1_, *X*_2_, and *X*_3_.

- Regression Model for Flexural Failure Load (*Y*_1_)

Equation (2) represents the regression model for the flexural failure load (*Y*_1_).

(2) $$Y_{1}=118.6+11.4X_{1}-3.833X_{2}+22.13X_{3}-0.795X_{1}^{2}-1.432X_{2}^{2}\;$$

The linear coefficient of *X*_1_ is positive (+11.4), indicating that the Flexural Failure Load (*Y*_1_) increases linearly as the Binder (*X*_1_) content increases. This is consistent with the general phenomenon of strength enhancement due to the strengthening of the alkali-activated matrix. Conversely, the linear coefficient of *X*_2_ is negative (−3.833), meaning that the flexural failure load decreases as the Perlite (*X*_2_) content increases. This reduction is attributed to perlite being a low-strength porous material; its increased proportion raises the internal porosity of the board, leading to a reduction in strength [27,28,29].

Although *X*_3_ (Wollastonite) has a linear term included, the removal of all interaction terms and the omission of its quadratic term ($X_{3}^{2}$) from the final fitted model indicate that *X*_3_ has only a limited linear effect on *Y*_1_. Thus, the primary changes in flexural failure load are determined by the Binder (*X*_1_) and Perlite (*X*_2_) contents.

- Regression Model for Moisture Content (*Y*_2_)

Equation (3) represents the regression model for the moisture content (*Y*_2_).

(3) $$Y_{2}=2.93-0.1432X_{1}+0.1467X_{2}-0.0256X_{3}+0.001795X_{1}^{2}+0.0171X_{2}^{2}-0.001778X_{1}X_{2}\;$$

Analysis of the second-order polynomial regression model for Moisture Content (*Y*_2_) reveals that changes in moisture content are primarily determined by the amounts of Binder (*X*_1_) and Perlite (*X*_2_) and their interaction, while the influence of Wollastonite (*X*_3_) is limited.

The linear coefficient of Perlite (*X*_2_) is positive (+0.1467), acting as the main factor driving the increase in moisture content. This is believed to be because perlite’s porous structure increases the overall porosity of the board, leading to greater water absorption. The linear coefficient of Binder (*X*_1_) is negative (−0.1432), counteracting the positive effect of perlite by reducing moisture content. This is attributed to the alkali-activated matrix becoming denser as the binder content increases, which reduces pores and suppresses water penetration [30,31].

The model for moisture content includes quadratic terms ($X_{1}^{2}$,$X_{2}^{2}$) and an interaction term (*X*_1_*X*_2_), explaining the composite effects. The quadratic effect coefficient for perlite ($X_{2}^{2}$) is positive (+0.0171), indicating a non-linear trend where the rate of moisture content increase accelerates as the perlite content rises. The interaction term (*X*_1_*X*_2_) coefficient is negative (−0.001778), suggesting that simultaneous increases in *X*_1_ and *X*_2_ interact to reduce the moisture content. This means the densifying effect of the binder partially suppresses perlite’s high hygroscopicity, controlling the moisture content increase.

The linear coefficient for Wollastonite (*X*_3_) is negative (−0.0256), and its absolute value is very small compared to the linear coefficients of *X*_1_ and *X*_2_ (around 0.14). This suggests that although *X*_3_ contributes towards reducing moisture content, its effect is marginal, and it is not a primary factor in determining the overall moisture content.

- Regression Model for Specific Gravity (*Y*_3_)

Equation (4) represents the regression model for the specific gravity (*Y*_3_).

(4) $$Y_{3}=1.275+0.01867X_{1}-0.0365X_{2}+0.000411X_{2}^{2}-0.000244X_{1}X_{2}$$

Analysis of the second-order polynomial regression model for Specific Gravity (*Y*_3_) shows that changes in specific gravity are primarily determined by the incorporation levels of Binder (*X*_1_) and Perlite (*X*_2_), and the influence of Wollastonite (*X*_3_) was found to be statistically non-significant.

The linear coefficient for Perlite (*X*_2_) is negative (−0.0365) and has the largest absolute value. This signifies that perlite is the strongest lightweight factor, most powerfully reducing the specific gravity as its incorporation increases. The linear coefficient for Binder (*X*_1_) is positive (+0.01867); as binder content increases, the proportion of the higher-density matrix component increases, thereby increasing the specific gravity.

The specific gravity model includes the quadratic term for binder ($X_{2}^{2}$) and the binder–perlite interaction term (*X*_1_*X*_2_). The coefficient for the quadratic effect of binder ($X_{2}^{2}$) is positive (+0.000411), indicating a non-linear trend where the rate of specific gravity increase accelerates as binder content increases, suggesting a pronounced density-increasing effect at higher binder levels. The interaction term (*X*_1_ *X*_2_) coefficient is negative (−0.000244). This implies that when both binder and perlite increase simultaneously, they interact to reduce the specific gravity, with perlite’s lightening effect partially counteracting the binder’s densifying effect.

Both the linear term and related interaction terms for Wollastonite (*X*_3_) were removed from the specific gravity model. This indicates that, within the tested range of wollastonite content, its effect on specific gravity (*Y*_3_) is statistically non-significant, which is consistent with the experimental results discussed in Section 3.1.

In summary, achieving the lightweight target (Specific Gravity 0.9) requires increasing the Perlite (*X*_2_) content. However, this simultaneously reduces the Flexural Failure Load (*Y*_1_). Therefore, the core challenge of the optimization lies in finding the critical equilibrium point between *X*_1_ and *X*_2_ that simultaneously satisfies all three responses (*Y*_1_, *Y*_2_, *Y*_3_).

#### 3.2.2. Analysis of Variance (ANOVA) Results

Table 6 presents the ANOVA results for the Flexural Failure Load (*Y*_1_). The ANOVA results for the Flexural Failure Load (*Y*_1_) indicated that the overall model is highly significant (*p*-value = 0). The most dominant factors explaining the flexural failure load were Binder (Adj SS = 32,512.5) and Perlite (Adj SS = 26,450), both having *p*-values of 0. Conversely, the linear effect of Wollastonite (*p*-value = 0.122) was not significant. In addition, the non-linear effects (quadratic terms) were significant (*p*-value = 0.011), particularly the Binder × Binder term (*p*-value = 0.009). This confirmed that the variation in flexural failure load with changes in binder content is non-linear. These findings collectively indicate that achieving the maximum flexural failure load requires finding an optimal combination point primarily centered on the contents of binder and perlite.

Table 7 presents the ANOVA results for the Moisture Content (*Y*_2_). The overall model is highly significant (*p*-value = 0). The most dominant factors explaining the variation in moisture content were Perlite (*p*-value = 0, Adj SS = 8) and Binder (*p*-value = 0, Adj SS = 6.845). Conversely, the linear effect of Wollastonite was not significant (*p*-value = 0.459). Furthermore, the non-linear effects (quadratic terms) were significant (*p*-value = 0.007), and notably, the two-way interaction between Binder and Perlite (*p*-value = 0.002) was highly significant. This highlights that controlling the moisture content is crucial and requires adjusting the contents of these two factors while considering their interconnectedness and non-linear relationship.

Table 8 presents the ANOVA results for the Specific Gravity (*Y*_3_). The ANOVA results for the Specific Gravity (*Y*_3_) indicated that the overall model is highly significant (*p*-value = 0). The most dominant factors explaining the variation in specific gravity were Perlite (*p*-value = 0, Adj SS = 0.19845) and Binder (*p*-value = 0, Adj SS = 0.1058). Notably, Perlite exhibited a greater influence on specific gravity than the Binder. In addition, the non-linear effects (quadratic terms) were significant (*p*-value = 0.001), primarily driven by the Perlite × Perlite term (*p*-value = 0.001), suggesting that the Perlite content non-linearly affects specific gravity. Furthermore, the Binder × Perlite interaction term (*p*-value = 0.013) was significant, highlighting the crucial combined role of these two factors in determining specific gravity.

In the ANOVA results for all three dependent variables (Flexural Failure Load, Moisture Content, and Specific Gravity), the Adj SS for Pure Error was 0 with a Degree of Freedom (DF) of 2. This is likely due to an insufficient number of replicated measurement points during the experimental design for statistical model verification, or the replicated measurements happened to match perfectly. Since the Adj MS for Pure Error became 0, the F-value and *p*-value for the Lack-of-Fit test, which assesses the adequacy of the model form, could not be calculated (indicated by *). Consequently, judging the statistical adequacy of the model form was limited for all three properties. However, the validity of the models was indirectly interpreted based on the highly significant statistical results (*p*-value = 0) observed for the main factors.

### 3.3. Optimal Condition Derivation via Response Surface Analysis

#### 3.3.1. Contour Plot Analysis

Contour Plots were generated using the statistically derived regression models (Section 3.2) to visually confirm the combined effects and interactions between the independent variables (*X*_1_, *X*_2_, *X*_3_) on the dependent variables (*Y*_1_, *Y*_2_, *Y*_3_).

**Figure 2:**
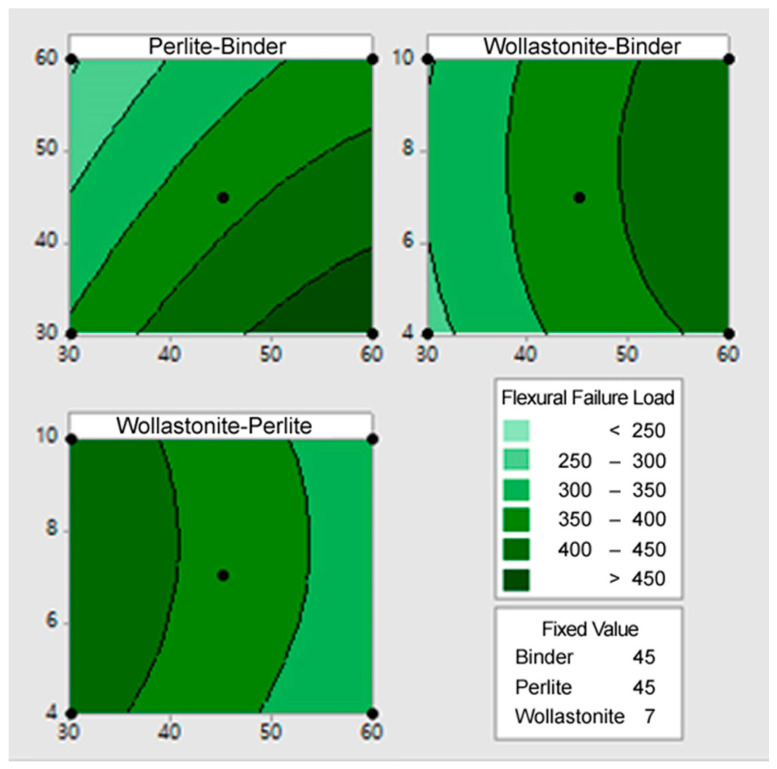
The contour plot for the flexural failure load (Y_1_).

Figure 2 illustrates the contour plot for the flexural failure load (*Y*_1_). The contour plot for the flexural failure load (*Y*_1_) clearly confirms that the optimal mix region for maximizing the flexural failure load (>450 N) is concentrated in the area where the Binder (*X*_1_) content is high (50~60%) and the Perlite (*X*_2_) content is low (30~40%). The contours run diagonally, confirming the strong antagonistic interaction between *X*_1_ and *X*_2_. This supports the finding that reducing the perlite content and increasing the binder content is the most effective strategy for improving the flexural failure load. Wollastonite (*X*_3_) showed high strength at intermediate levels (6~8%). Overall, maximizing the flexural failure load requires maximizing binder content, minimizing perlite content, and applying an appropriate amount of wollastonite within the intermediate to high range, taking into account the combination of *X*_1_ and *X*_2_.

**Figure 3:**
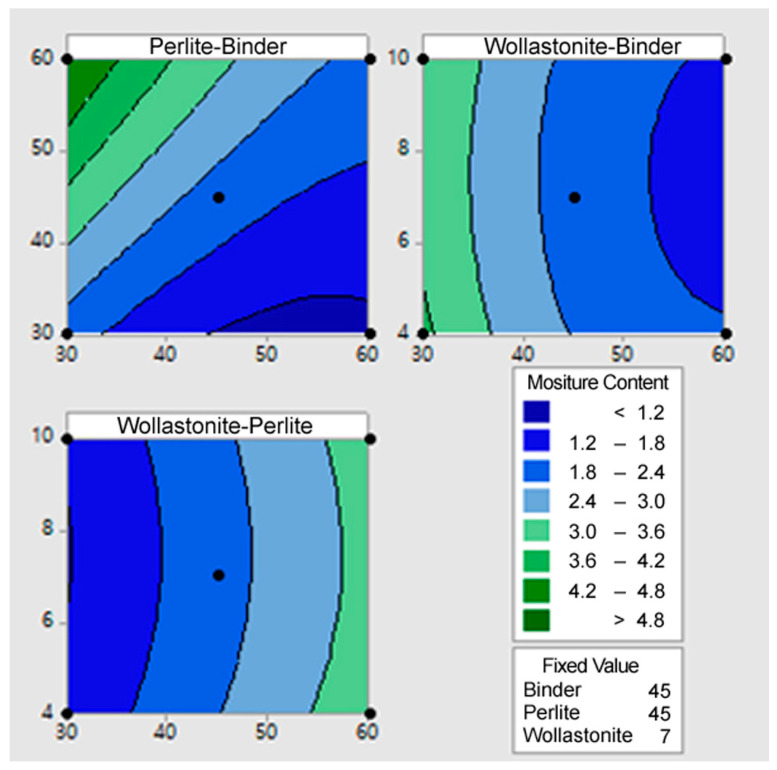
The contour plot for the moisture content (Y_2_).

Figure 3 illustrates the contour plot for the moisture content (*Y*_2_). The contour plot for moisture content (*Y*_2_) clearly illustrates the optimal mix conditions for minimizing moisture content (the dark blue region of 1.2~1.8% or less). In all graphs, the moisture content increases most sharply as the perlite content increases, indicating that the porous nature of perlite is the primary cause of high moisture absorption. Therefore, controlling the perlite content at the lowest level (30~40%) is essential for moisture content minimization. Conversely, moisture content tends to decrease when the binder content is maintained high (50~60%) and the wollastonite content is set high (8~10%). This is because the binder and wollastonite densify the mixture’s microstructure, reducing pores and lowering water absorption capacity. Collectively, the optimal mix conditions for minimizing moisture content are determined at the point where perlite content is minimized, and binder and wollastonite contents are maximized to increase the overall density of the mixture. This pattern is consistent with the conditions that maximize the flexural failure load.

**Figure 4:**
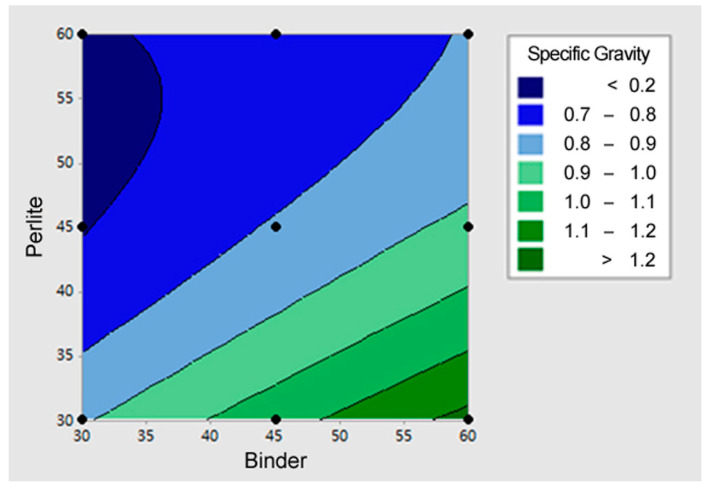
The contour plot for the specific gravity (Y_3_).

Figure 4 illustrates the contour plot for the specific gravity (*Y*_3_). The contour plot for specific gravity (*Y*_3_) clearly shows the conflicting effects of perlite (the lightweight material) and binder (the density-increasing material). The minimum specific gravity area (dark blue area <0.7) appeared in the combination where the Perlite content was high (55~60%) and the Binder content was low (30~35%). This indicates that to minimize specific gravity, the content of perlite—the strongest contributor to density reduction—must be maximized, while the content of binder—which increases density—must be minimized. However, since this combination corresponds to the condition that minimizes the flexural failure load, it is crucial during the final mix design determination to find the optimal proportion that minimizes specific gravity while simultaneously satisfying the required minimum strength criteria.

#### 3.3.2. Response Surface Analysis (3D Plots)

**Figure 5:**
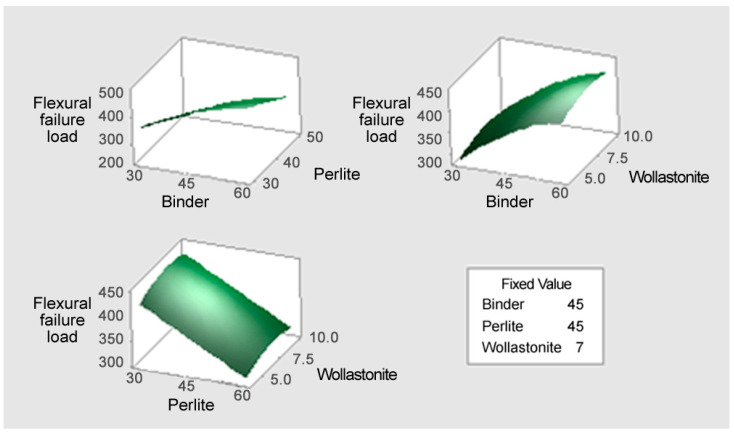
The response surface plot for the flexural failure load (Y_1_).

Figure 5 illustrates the response surface plot for the flexural failure load (*Y*_1_). The optimal conditions for maximizing the flexural failure load are clearly evident when the binder content is high and the perlite content is low. The Binder–Perlite surface shows a steep gradient where the flexural failure load rapidly decreases with increasing perlite content and increases with increasing binder content. Furthermore, the Binder–Wollastonite surface shows that the flexural failure load increases with higher binder content, while wollastonite exhibits a convex, non-linear shape along its axis, with the highest flexural failure load found around the center (7.5%). Therefore, to maximize the flexural failure load, it is necessary to set the binder content at the maximum level, the perlite content at the minimum level, and the wollastonite content at the optimal intermediate point, taking into account their combined effects.

**Figure 6:**
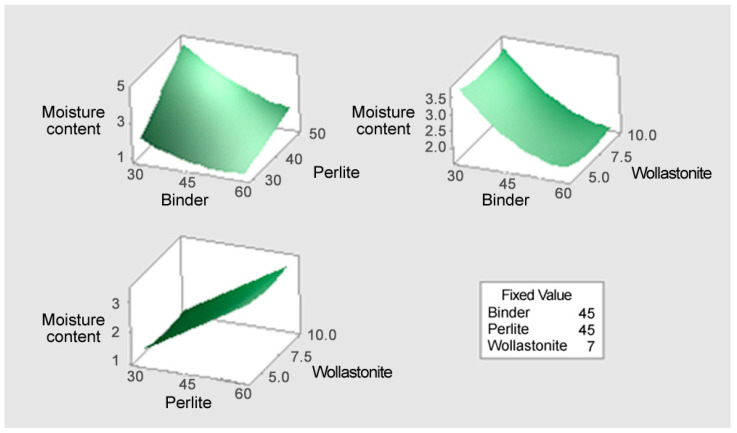
The response surface plot for the moisture content (Y_2_).

Figure 6 illustrates the response surface plot for the moisture content (*Y*_2_). The conditions that minimize the moisture content (the lowest point on the surface) occur when the binder content is high and the perlite content is low. The Binder–Perlite surface shows a tendency for the moisture content to increase sharply with higher perlite content, and decrease with higher binder content, indicating that perlite is the most dominant factor in increasing moisture absorption. Additionally, both the Binder–Wollastonite and Perlite–Wollastonite surfaces show a tendency for moisture content to be minimized when binder content is high and perlite content is low. Consequently, to minimize moisture content, it is essential to set the perlite content at the minimum level and the binder and wollastonite contents at high levels to ensure the density of the mixture.

**Figure 7:**
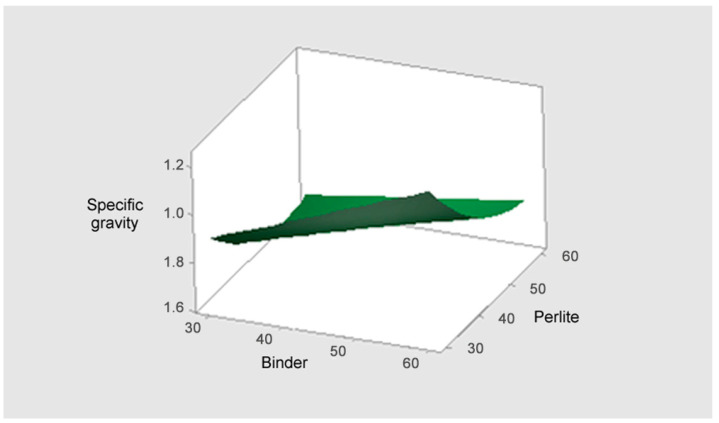
The response surface plot for the specific gravity (Y_3_).

Figure 7 illustrates the response surface plot for the specific gravity (*Y*_3_). The response surface clearly shows a sharp decrease in specific gravity as perlite content increases and an increase in specific gravity as binder content increases. Therefore, the conditions that minimize specific gravity (the lowest point on the surface) occur when the Perlite content is maximized (55~60%) and the Binder (solidifying agent) content is minimized (30~35%). This result implies that perlite is the most powerful factor in density reduction. While minimizing specific gravity requires maximizing perlite and minimizing binder, this combination is diametrically opposed to the conditions that maximize the flexural failure load. Thus, securing the mix proportion while ensuring the minimum required flexural failure load is critical.

### 3.4. Optimal Mix Proportion Derivation via Response Surface Analysis

Based on the response surface analysis results for the lightweight board’s flexural failure load, moisture content, and specific gravity, a response optimizer simulation was conducted using Minitab 18. The optimization variables were set to simultaneously achieve conflicting goals, as shown in Table 9.

**Figure 8:**
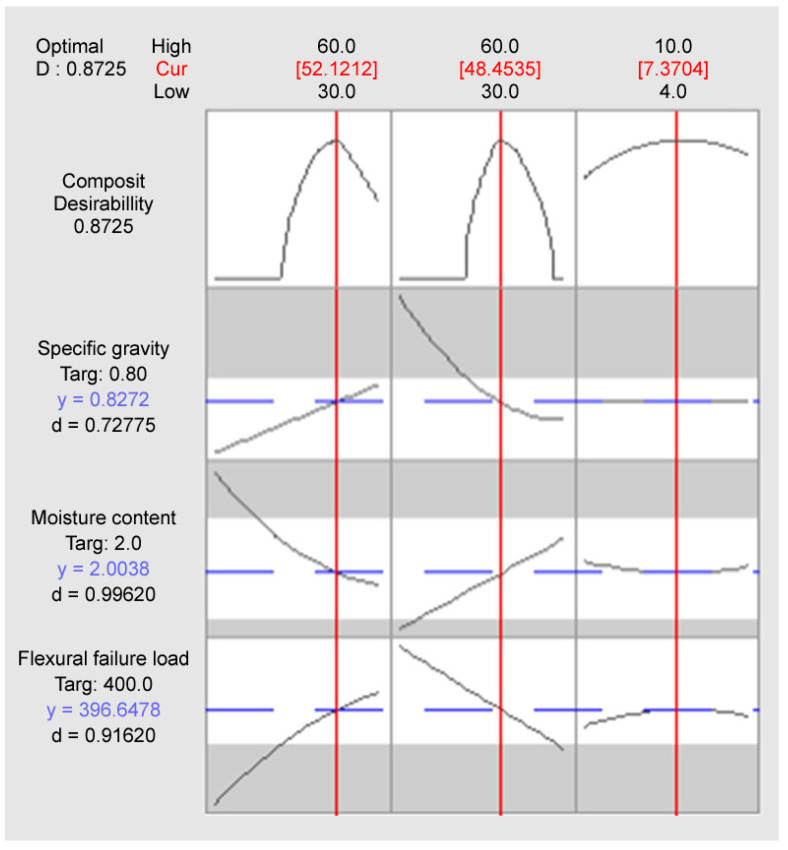
Results of the response optimizer simulation.

Figure 8 and Table 10 present the results of the response optimizer simulation.

The simulation successfully derived the optimal mix conditions that simultaneously satisfy all three set goals: Binder 52.12%, Perlite 48.45%, and Wollastonite 7.37%. The Composite Desirability (D) for this blend was 0.8725, indicating a very high level of overall satisfaction and successful fulfillment of the complex requirements within the set acceptance ranges.

Specifically, Moisture Content showed the highest individual desirability (0.99620), with a predicted value of 2.0038% which is nearly perfect agreement with the 2.0% target. Flexural Failure Load also achieved a high desirability of 0.91620, with a predicted value of 396.6478 N, which is very close to the 400 N target. The predicted Specific Gravity was 0.8272, slightly higher than the 0.80 target, but it yielded a desirability of 0.72775 and successfully met the lightweight goal within the set upper limit (0.9).

Furthermore, the statistical predictability of the optimal point was evaluated, as shown in Table 11. The 95% Confidence Interval (CI) provides the estimated range for the true mean of the response, while the 95% Prediction Interval (PI) estimates the range where a single future observation is likely to fall. For instance, the predicted flexural failure load of 396.82 N is highly likely to fall between 371.10 N and 422.54 N in a future single experiment, confirming the model’s reliability in predicting performance near the optimal point.

## 4. Experimental Verification of Response Optimization Results

To verify the validity of the optimal mix proportion derived from the response optimization simulation (Composite Desirability D = 0.8725) and the accuracy of the model’s predicted values, actual experiments were conducted. The confirmed weight proportions for the verification sample were Binder 52.12%, Perlite 48.45%, and Wollastonite 7.37%. Lightweight board samples were manufactured using this mix ratio, following the identical procedures detailed in Section 2.3, and subjected to physical property testing.

Table 12 compares the test results for the optimal mix sample with the predicted values from the Response Optimization model. The experimental values for all three response variables were found to be very close to the predicted values, demonstrating the high predictive precision of the model.

For the flexural failure load, the experimental value (408.54 N) was 11.72 N higher than the predicted value, with a percentage error of 2.87%. This high level of accuracy confirmed that the strength target (400 N) was successfully surpassed.

For the moisture content, the experimental value (2.15%) was 0.15% higher than the predicted value, resulting in a percentage error of 6.98%. Although this error was slightly higher compared to the flexural failure load and specific gravity, the measured value was still well within the set upper limit of 3.0%, thus sufficiently satisfying the target.

The specific gravity showed the lowest percentage error of 2.35%, with the experimental value (0.85 g/cm^3^) being only 0.02 g/cm^3^ higher than the predicted value (0.83 g/cm^3^). This result validates that the model’s prediction for the lightweight goal was highly accurate.

The overall results imply that the response surface model, which includes non-linear and interaction terms, accurately reflects the complex interplay of the factors. Therefore, the derived optimal mix proportion is definitively verified as the most reliable blend condition for simultaneously meeting the conflicting goals of maintaining strength, reducing moisture content, and achieving lightweight properties.

## 5. Conclusions

This study aimed to establish the optimal mix proportion for manufacturing high-performance lightweight boards utilizing an eco-friendly binder system based on Ground Granulated Blast-furnace Slag (GGBFS) and waste rock wool, and to statistically verify its effectiveness.

The Response Surface Methodology (RSM) analysis confirmed that binder and perlite were the most dominant factors influencing all three properties: flexural failure load, moisture content, and specific gravity. Critically, maximizing the flexural failure load and minimizing the specific gravity were found to demand conflicting conditions regarding the contents of binder and perlite. While the linear effect of wollastonite was marginal, its non-linear tendency to exhibit maximum strength at an intermediate level demonstrated the necessity of considering the complex interactions of all factors when searching for the optimal mix point.

A response optimization simulation successfully derived an optimal mix point that simultaneously satisfied the three conflicting targets: a flexural failure load of 400 N, a moisture content of 2.0%, and a specific gravity of 0.80. The simulation yielded a very high Composite Desirability (D) of 0.8725, and the final optimal weight proportions were determined to be Binder 52.12%, Perlite 48.45%, and Wollastonite 7.37%. The model’s predicted values at this optimal blend were a flexural failure load of 396.82 N, moisture content of 2.00%, and specific gravity of 0.83 g/cm^3^, nearly perfectly meeting the moisture content target and closely approximating the flexural failure load target.

The reliability of the RSM model was definitively established through the experimental verification of the derived optimal mix proportion. The actual measured experimental values were a flexural failure load of 408.54 N, moisture content of 2.15%, and specific gravity of 0.85 g/cm^3^. Compared to the predicted values, all responses showed a low percentage error, falling within 7%. Notably, the flexural failure load successfully exceeded the 400 N target, validating the most critical goal of securing minimum strength.

Therefore, the optimal mix proportion presented in this study can be utilized as reliable foundational design data necessary for the development of GGBFS-based eco-friendly lightweight boards. This work is expected to lay the groundwork for future commercialization research into construction materials with complex performance capabilities. Furthermore, by maximizing the use of waste materials, this work inherently possesses economic advantages and is expected to lay the groundwork for future commercialization research, enabling a secondary, economy-based optimization where raw material cost can be incorporated as a variable to ensure market competitiveness.

## Figures and Tables

**Table 1:** Composition and Proportion of the Binder System.

Water to Binder Ratio	Binder Material Weight Ratio (%)			Alkali Activator	
	GGBFS (A)	Waste Rock Wool (B)	Anhydrous Gypsum	NaOH	Na_2_CO_3_
40	70	20	10	3% of (A+B) weight	5% of (A+B) weight

**Table 2:** Chemical Composition of the Waste Mineral Wool.

Composition (%)								
SiO_2_	CaO	Al_2_O_3_	Fe_2_O_3_	MgO	K_2_O	Na_2_O	SO_3_	TiO_2_
33.32	21.24	15.77	8.57	8.02	0.72	1.51	0.47	0.39

**Table 3:** Experimental factors and levels for response surface methodology.

Code	Independent Variable (Experimental Factor)	Factor Range (wt%)	Coded Level (−1)	Coded Level (0)	Coded Level (+1)
*X*_1_	Binder	30~60	30	45	60
*X*_2_	Expanded Perlite	30~60	30	45	60
*X*_3_	Wollastonite	4~10	4	7	10

**Table 4:** Mix Design Runs for the Box–Behnken Design (BBD) Experiment.

Run No.	Code	Binder (*X*_1_, wt%)	Perlite (*X*_2_, wt%)	Wollastonite (*X*_3_, wt%)	Note
1	(0, 0, 0)	45	45	7	Center point
2	(+1, −1, 0)	60	30	7	Factor point
3	(+1, +1, 0)	60	60	7	Factor point
4	(−1, 0, +1)	30	45	10	Factor point
5	(−1, −1, 0)	30	30	7	Factor point
6	(0, −1, −1)	45	30	4	Factor point
7	(0, +1, +1)	45	60	10	Factor point
8	(0, 0, 0)	45	45	7	Center point
9	(0, −1, 1)	45	30	10	Factor point
10	(−1, 0, −1)	30	45	4	Factor point
11	(0, 0, 0)	45	45	7	Center point
12	(+1, 0, −1)	60	45	4	Factor point
13	(+1, 0, +1)	60	45	10	Factor point
14	(0, +1, −1)	45	60	4	Factor point
15	(−1, +1, 0)	30	60	7	Factor point

**Table 5:** Experimental Results of Physical Properties.

Run No.	Binder (*X*_1_, wt%)	Perlite (*X*_2_, wt%)	Wollastonite (*X*_3_, wt%)	Flexural Failure Load (*Y*_1_, N)	Moisture Content (*Y*_2_, %)	Specific Gravity (*Y*_3_)
1	45	45	7	380.3	2.2	0.80
2	60	30	7	479.7	1.2	1.21
3	60	60	7	371.0	2.4	0.82
4	30	45	10	289.7	3.8	0.72
5	30	30	7	361.0	1.9	0.84
6	45	30	4	440.3	1.3	1.08
7	45	60	10	320.7	3.2	0.74
8	45	45	7	381.0	2.2	0.81
9	45	30	10	430.0	1.3	1.12
10	30	45	4	270.7	3.9	0.71
11	45	45	7	380.0	2.2	0.80
12	60	45	4	410.0	1.7	0.94
13	60	45	10	430.3	1.6	0.87
14	45	60	4	300.0	3.4	0.76
15	30	60	7	260.0	4.7	0.66

**Table 6:** ANOVA Results for Flexural Failure Load (*Y*_1_).

Source	DF	Adj SS	Adj MS	F-Value	*p*-Value
Model	5	60,966	12,193.2	113.45	0
Linear	3	59,275	19,758.3	183.83	0
Binder	1	32,512.5	32,512.5	302.5	0
Perlite	1	26,450	26,450	246.1	0
Wollastonite	1	312.5	312.5	2.91	0.122
Quadratic	2	1691	845.5	7.87	0.011
Binder × Binder	1	1188	1188	11.05	0.009
Wollastonite × Wollastonite	1	616.6	616.6	5.74	0.04
Error	9	967.3	107.5		
Lack-of-Fit	7	967.3	138.2	*	*
Pure Error	2	0	0		
Total	14	61,933.3			

* Indeterminate (Due to Adj MS for Pure Error equaling zero).

**Table 7:** ANOVA Results for Moisture Content (*Y*_2_).

Source	DF	Adj SS	Adj MS	F-Value	*p*-Value
Model	6	16.1691	2.69485	81.59	0
Linear	3	14.865	4.955	150.02	0
Binder	1	6.845	6.845	207.24	0
Perlite	1	8	8	242.21	0
Wollastonite	1	0.02	0.02	0.61	0.459
Quadratic	2	0.6641	0.33205	10.05	0.007
Binder × Binder	1	0.6058	0.60577	18.34	0.003
Wollastonite × Wollastonite	1	0.0879	0.08791	2.66	0.141
2-Way Interaction	1	0.64	0.64	19.38	0.002
Binder × Perlite	1	0.64	0.64	19.38	0.002
Error	8	0.2642	0.03303		
Lack-of-Fit	6	0.2642	0.04404	*	*
Pure Error	2	0	0		
Total	14	16.4333			

* Indeterminate (Due to Adj MS for Pure Error equaling zero).

**Table 8:** ANOVA Results for Specific Gravity (*Y*_3_).

Source	DF	Adj SS	Adj MS	F-Value	*p*-Value
Model	4	0.348293	0.087073	65.96	0
Linear	2	0.30425	0.152125	115.25	0
Binder	1	0.1058	0.1058	80.15	0
Perlite	1	0.19845	0.19845	150.34	0
Quadratic	1	0.031943	0.031943	24.2	0.001
Perlite × Perlite	1	0.031943	0.031943	24.2	0.001
2-Way Interaction	1	0.0121	0.0121	9.17	0.013
Binder × Perlite	1	0.0121	0.0121	9.17	0.013
Error	10	0.0132	0.00132		
Lack-of-Fit	8	0.0132	0.00165	*	*
Pure Error	2	0	0		
Total	14	0.361493			

* Indeterminate (Due to Adj MS for Pure Error equaling zero).

**Table 9:** Optimization Goals for Response Variables.

Response	Goal	Lower Limit	Target	Upper Limit	Weight	Importance
Flexural Failure Load	Target	360	400	480	1	1
Moisture Content	Target	1.2	2.0	3.0	1	1
Density	Target	0.6	0.8	0.9	1	1

**Table 10:** Optimal Mix Ratio and Predicted Responses (RSM Model).

Factor	Optimal Mix Ratio	Response	Target	Predicted Value (y)	Desirability (d)
Binder	52.1212	Moisture Content	2.0	2.0038	0.99620
Perlite	48.4535	Flexural Failure Load	400.0	396.6478	0.91620
Wollastonite	7.3704	Density	0.80	0.8272	0.72775

**Table 11:** Statistical Metrics for Predicted Optimal Responses.

Response	Fitted Value	SE Fit	95% CI	95% PI
Flexural Failure Load	396.82	4.67	(386.26, 407.37)	(371.10, 422.54)
Moisture Content	2.0038	0.0824	(1.8139, 2.1940)	(1.5438, 2.4641)
Density	0.8272	0.0148	(0.7942, 0.8602)	(0.7398, 0.9146)

**Table 12:** Comparison of Predicted and Verified Optimal Properties.

Category	Flexural Failure Load (N)	Moisture Content (%)	Density (g/cm^3^)
Predicted Value	396.82	2.00	0.83
Experimental Value	408.54	2.15	0.85
Deviation (Absolute)	11.72	0.15	0.02

## Data Availability

The original contributions presented in this study are included in the article. Further inquiries can be directed to the corresponding author.
